# Bronchogenic cysts mimicking thymoma in the anterior mediastinum

**DOI:** 10.1002/rcr2.583

**Published:** 2020-05-12

**Authors:** Jonggeun Lee, Jee Won Chang

**Affiliations:** ^1^ Department of Thoracic and Cardiovascular Surgery, School of Medicine Jeju National University Hospital, Jeju National University Jeju South Korea

**Keywords:** Bronchogenic cyst, mediastinal neoplasms, thoracic surgery, thymoma, video‐assisted

## Abstract

Bronchogenic cysts are commonly located in the middle mediastinal compartment as fluid‐filled cysts and thymoma is one of the most common neoplasms in the anterior mediastinum in adult cases. For two cases of asymptomatic, incidentally discovered anterior mediastinal soft tissue mass in adults, we planned to perform complete thymectomy with minimally invasive techniques based on the guidelines of International Thymic Malignancy Interest Group. Their pathological finding revealed cystic lesions lined by ciliated epithelium and this supported the diagnosis of bronchogenic cyst rather than thymic neoplasm. We report the two cases of resected bronchogenic cysts which were in the unusual location of anterior mediastinum with uncommon radiological feature.

## Graphical abstract

We present two cases of surgically resected bronchogenic cysts in the anterior mediastinum mimicking thymoma.

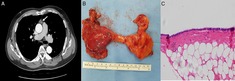

## Introduction

Primary mediastinal masses are heterogeneous and have their own predilections for a specific compartment. Masses that are usually located in the anterior compartment of mediastinum are thymoma, germ cell tumours, or lymphoma, and thymoma is one of the most common neoplasm in adult cases [1]. On the other hand, mediastinal cysts arising from viscera, neurogenic structures, or mesenchymal tissues are found in the middle or posterior compartment [1].

Bronchogenic cyst is mostly found in the middle mediastinal compartment as a well‐circumscribed, fluid‐filled lesion on computed tomography (CT) scan. However, it can be misdiagnosed as other types of mediastinal mass if it is discovered in unusual location with uncommon radiological characteristics [2].

We report two cases of surgically treated bronchogenic cyst which were located in the anterior mediastinum with soft tissue density on CT scan.

## Case Report

### Patient 1

**Figure 1:**
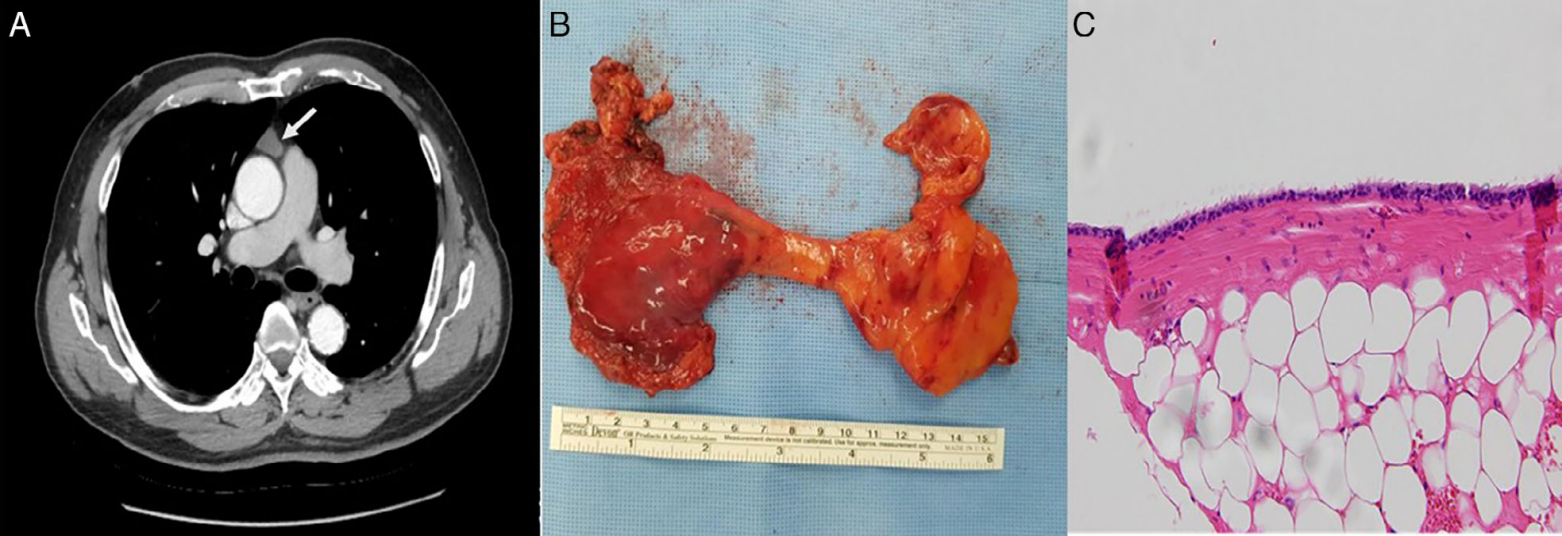
Contrast‐enhanced chest computed tomography (CT) scan shows a 2 × 1.2 cm sized, well‐defined enhancing soft tissue mass (A, arrow). Complete thymectomy including mediastinal fibroadipose tissue (B) revealed the cyst which is lined with pseudostratified ciliated columnar epithelium (C) (haematoxylin and eosin (H&E), 200×).

A 64‐year‐old male patient with previous history of hypercholesterolaemia presented to our outpatient clinic due to incidentally discovered mass in the anterior mediastinum on chest CT for routine check‐up. He did not complain of myasthenic symptoms or chest pain and his chest radiography showed no significant abnormality. Contrast‐enhanced CT scan showed a 1.4 × 1.8 × 2.6 cm sized, well‐defined mass with homogeneous attenuation (Hounsfield units (HU) of 42) (Fig. 1A). The radiological findings were suggestive of thymic epithelial tumour rather than cystic lesions and we decided to perform thoracoscopic complete thymectomy. Under general anaesthesia, the patient was placed in the left lateral decubitus position and left‐sided single lung ventilation was done for adequate visualization. Resection of thymus and surrounding mediastinal fat in the anterior mediastinum was done. Dissection and visualization of brachiocephalic vein and both phrenic nerves was performed for complete thymectomy. The specimen was retrieved and a 24‐Fr chest tube was placed in the right pleural cavity. The post‐operative course was uneventful and the patient was discharged on the third post‐operative day. Gross finding of the specimen showed a 2.0‐cm‐sized unilocular cystic structure within involuted thymus tissue without any communication with airway or oesophagus (Fig. 1B). Pathological examination revealed that a thin‐walled cyst was surrounded by fibroadipose tissue and filled with whitish milky fluid. Histologically, the cyst was lined with ciliated columnar bronchial epithelium and these findings supported the diagnosis of bronchogenic cysts (Fig. 1C).

### Patient 2

**Figure 2:**
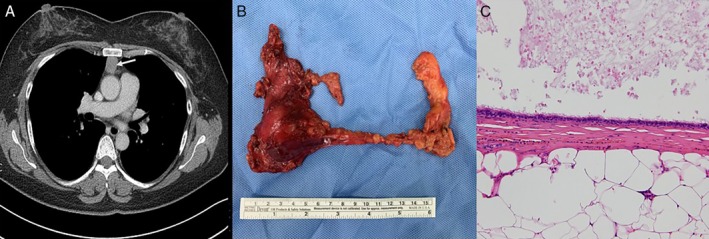
Contrast‐enhanced chest computed tomography (CT) scan shows a 2.2 × 2.0 cm sized, well‐circumscribed soft tissue mass (A, arrow). Complete thymectomy (B) revealed the unilocular cyst with ciliated columnar and flattened cuboidal epithelial cells (C) (haematoxylin and eosin (H&E), 200×).

A 44‐year‐old female patient was referred for evaluation of incidentally discovered anterior mediastinal mass from health check‐up centre. Her medical history was unremarkable except previous uterine myomectomy and she did not complain of any symptoms related to myasthenia gravis. There were no significant abnormalities on physical examination or in the laboratory tests. Contrast‐enhanced chest CT scan revealed an anterior mediastinal mass measuring 2.2 × 2.0 × 3.6 cm with soft tissue attenuation (HU of 60) (Fig. 2A). Thoracoscopic complete thymectomy was performed in the same ways as described in patient 1. She was discharged on the fifth post‐operative day without any post‐operative complications. The specimen showed that it was surrounded by mediastinal fat tissue grossly and was not communicated with respiratory tract (Fig. 2B). The histological examination revealed unilocular cyst surrounded by thymic tissue and filled with yellow mucoid materials. It was lined by flattened cuboidal cells and ciliated epithelium and these were appropriate for the bronchogenic cyst (Fig. 2C).

## Discussion

Bronchogenic cysts are congenital anomaly derived from abnormal germination of the embryonic foregut during the first 16 weeks of gestation [2] and finally differentiated into fluid‐filled pouch instead of normal bronchial development. Bronchogenic cysts are one of the most common types of mediastinal cysts and account for 10–15% of all primary mediastinal masses [3]. With the development of advanced diagnostic tools and regular medical check‐up, the identification of asymptomatic bronchogenic cysts is expected to be increased. Bronchogenic cysts are likely to be located in the middle or posterior mediastinum because they grow along the tracheobronchial tree from the perspectives of embryologic development [4, 5, 6].

Intrathymic or anterior mediastinal bronchogenic cysts are extremely rare [7, 8] and Taniwaki et al. published the first case of bronchogenic cyst within thymus in 1997 [7]. Table 1 presents the reported cases of bronchogenic cysts located in the thymus and perithymic fat tissue during recent 20 years and most of the reported cases of anterior mediastinal or intrathymic bronchogenic cysts presented as well‐circumscribed, fluid‐filled lesions on the chest CT [8, 9, 10, 11, 12, 13]. Unusual locations of bronchogenic cyst other than anterior mediastinum were reported in skin, subcutaneous tissue, neck, mesentery of bowel, or intramedullary part of the spine [14].

**Table 1:** Reported cases of resected anterior mediastinal bronchogenic cysts using minimally invasive techniques.

Published year	Authors	Age	Sex	Presentation	Radiological features
2020	Tamaki et al. [9]	61	F	Asymptomatic	Low signal in T1‐weighted image, high signal in T2‐weighted image on MRI (cystic)
2018	Hamouri et al. [8]	50	F	Asymptomatic	Oval‐shaped, enhancing soft tissue mass (HU: 55)
2017	Caterino et al. [10]	66	M	Asymptomatic	Soft tissue attenuation
2015	Mega [11]	55	F	Paroxysmal supraventricular tachycardia	Non‐enhancing, fluid‐filled lesion with calcification
2014	Hashimoto et al. [12]	66	M	Asymptomatic	Two anterior mediastinal lesions—cystic and nodular, respectively
1999	Takasuna et al. [13]	57	F	Asymptomatic	Cystic attenuation
		71	M	Asymptomatic	Cystic attenuation

HU, Hounsfield unit; MRI, magnetic resonance imaging.

Cardinale et al. [2] suggested the diagnostic criteria for bronchogenic cysts based on the location (middle mediastinum) and density expressed by HU (0–20) on CT scan. However, radiological heterogeneity of bronchogenic cysts has also been noted because the contents of bronchogenic cysts vary depending on the proportion of water and protein in them. One of the reports demonstrated that 25 of 58 (43.1%) pathologically confirmed bronchogenic cysts showed soft tissue attenuation (above 20 HU) at CT scan [14]. The cases included in this study also presented that intrathymic bronchogenic cysts had HU of 42 and 60, respectively, and such characteristics might be related to gross findings of milky or mucoid content of cysts. Therefore, most probable diagnosis was thymoma rather than mediastinal cysts preoperatively.

As long as surgery of thymoma is concerned, the International Thymic Malignancy Interest Group (ITMIG) recommended that patients should undergo a complete thymectomy including mass itself, surrounding thymus, and mediastinal fat even though they did not have myasthenia gravis [15]. According to the recommendations of ITMIG, such surgical principles should not be compromised because of oncological concerns when minimally invasive surgical techniques were applied. Considering the location and radiological characteristics of the cases in this study, we should plan complete thymectomy rather than mass excision preoperatively based on the ITMIG guidelines. Preoperative pathological diagnosis using fine needle aspiration (FNA) biopsy or needle core biopsy might be controversial even in the cases suspicious of thymic cyst because most prominent histopathological difference between thymic cyst and bronchogenic cyst is cilia of lining epithelial cells [5].

In summary, this study presented two cases of resected anterior mediastinal bronchogenic cyst using minimally invasive techniques. Surgical resection with the intention of complete thymectomy is recommended for accurate pathological diagnosis and curative treatment.

### Disclosure Statement

Appropriate written informed consent was obtained for publication of this case report and accompanying images.
